# Regulation of CYP450 and drug transporter mediated by gut microbiota under high-altitude hypoxia

**DOI:** 10.3389/fphar.2022.977370

**Published:** 2022-09-15

**Authors:** Xue Bai, Jianxin Yang, Guiqin Liu, Junbo Zhu, Qian Wang, Wenqi Gu, Linli La, Xiangyang Li

**Affiliations:** ^1^ Research Center for High Altitude Medicine, Qinghai University Medical College, Xining, China; ^2^ State Key Laboratory of Plateau Ecology and Agriculture, Qinghai University, Xining, China; ^3^ Medical College, Qinghai University Medical College, Xining, China

**Keywords:** cytochrome P450, drug metabolism, drug transporter, gut microbiota, high-altitude hypoxia

## Abstract

Hypoxia, an essential feature of high-altitude environments, has a significant effect on drug metabolism. The hypoxia–gut microbiota–CYP450/drug transporter axis is emerging as a vital factor in drug metabolism. However, the mechanisms through which the gut microbiota mediates the regulation of CYP450/drug transporters under high-altitude hypoxia have not been well defined. In this study, we investigated the mechanisms underlying gut microbial changes in response to hypoxia. We compared 16S ribosomal RNA gene sequences of the gut microbiota from plain and hypoxic rats. As a result, we observed an altered gut microbial diversity and composition in rats under hypoxia. Our findings show that dysregulated gut microbiota changes CYP3A1 and MDR1 expressions in high-altitude hypoxic environments. Thus, our study reveals a novel mechanism underlying the functioning of the hypoxia–gut microbiota–CYP450/drug transporter axis.

## Introduction

In high-altitude hypoxic environments, low pressure, low oxygen level, and strong radiation are essential characteristics, with low oxygen level being a key factor affecting human life activities (Li et al., 2012). Under hypoxic conditions, the circulatory system, endocrine system, nervous system, and metabolism undergo significant functional changes (Eide and Asplund, 2012; Zafren, 2014). Meanwhile, hypoxia triggers a series of both physiological and pathological changes in the body, which have specific effects on drug pharmacokinetics through regulating the activity and expression of cytochrome P450 (CYP450) and drug transporter, further influencing therapeutic outcomes (Zhou et al., 2018). Importantly, these results indicate that the metabolic profiling of drugs is obviously different between people living in plains and plateaus.

In recent years, hundreds of thousands of lowlanders have traveled to plateau areas for business, athletic training, recreation, and military activities (Zhou et al., 2018). Upon entering the plateau, a considerable proportion of these people may experience acute mountain sickness or other altitude-related illnesses (Li et al., 2019). Moreover, chronic hypoxic environments in high-altitude areas induce polycythemia and cardiovascular diseases (Imray et al., 2010). Medical assistance is often limited, and there is a lack of guidance and recommendations for the use of clinical medications in plateau areas. Consequently, the dosage and frequency of medications still follow recommendations for their use in plain areas, resulting in an inability to guarantee rational and safe medication for lowland populations in plateau areas.

The metabolism of most drugs is altered under hypoxia, with significant changes in pharmacokinetic parameters, mainly manifested as a significant prolongation of half-life and mean residence time and decreased clearance rate. Significant changes in CYP450 and drug transporter expression have also been observed in high-altitude hypoxic environments (Bai et al., 2022). Research studies have shown that acute hypoxia is linked to the downregulation of CYP1A2, CYP2B4, and CYP2C16 expressions and upregulation of CYP3A6 expression in rabbits (Fradette et al., 2007). Our previous study showed that the activity and expression of CYP1A1 and CYP2E1 are downregulated and the expression of CYP2D1 is upregulated under high-altitude hypoxia (Li et al., 2014). Interestingly, hypoxia seems to have a mixed influence on drug transporter expression. When subjected to hypoxic environments for 48 h, the expression of multiple drug resistance protein 1 (MDR1) significantly increased by 85% in rabbits (Li et al., 2016). In another study, hypoxic treatment for 72 h downregulated the protein and mRNA expressions of MDR1 by 71.3% and 51.0% in the small intestine and upregulated it by 1.33-fold and 1.15-fold, respectively, in the liver tissue (Luo et al., 2016). Conflicting results have also been reported for other drug transporters, such as the expression of multidrug-resistance-associated protein 2 (MRP2), peptide transporter 1 (PEPT1), organic anion transporter polypeptide 1B1 (OATP1B1), and organic cation transporter 1 (OCT1) (du Souich and Fradette, 2011; Park et al., 2012; Wojtal et al., 2014).

Gut microbiota is a complex ecosystem formed by trillions of microorganisms inhabiting the gastrointestinal tract, affecting normal physiology and disease susceptibility (Lozupone et al., 2012). The gut microbiota may directly utilize distinct microbial enzymes and metabolites to participate in drug metabolism and may also indirectly modify the biotransformation of drugs by regulating CYP450 and drug transporter (Carmody and Turnbaugh, 2014; Li et al., 2016). Selwyn et al. (2015) conducted in-depth transcriptomic studies which identified that the mRNA expression of CYP2A5, CYP2A22, OATP1B2, and MRP2 increased, whereas that of CYP3A11 decreased, in germ-free mice.

To date, few reports have been published on the factors affecting changes in CYP450 and drug transporter expression in high-altitude hypoxic environments. In addition, direct evidence concerning gut microbiota dysregulation mediating CYP450 and drug transporter expression under high-altitude hypoxia is still lacking (Bai et al., 2022). Consequently, in this study, we used the unique geographical conditions of the Qinghai–Tibet Plateau to investigate the joint effects of gut microbiota, drug metabolism, and a high-altitude hypoxic environment. Changes in gut microbiota were analyzed, and the regulation of CYP450 (CYP1A2, CYP2B1, CYP2C11, CYP2E1, and CYP3A1) and drug transporters (MDR1, MRP2, BCRP, OATP2B1, OCT1, and PEPT1) by the gut microbiota was explored in high-altitude hypoxic environments.

## Materials and methods

### Chemicals

Vancomycin (lot: C11498870), neomycin sulfate (lot: C11606396), metronidazole (lot: C111653035), and ampicillin (lot: C10868057) were purchased from Shanghai Macklin Biochemical (Shanghai, China). HiPure Stool DNA Kits (lot: D3141) were obtained from Guangzhou Magen Biological Technology (Guangzhou, China). Anti-mouse β-actin (1:500 dilution, Abcam, lot: ab8226), anti-mouse CYP1A2 (1:1000 dilution, Abcam, lot: ab22717), anti-mouse CYP2B1 (1:1000 dilution, Thermo Fisher, lot: MA5-25882), anti-rabbit CYP2C11 (1:1000 dilution, Biorbyt, lot: orb5951), anti-rabbit CYP2E1 (1:1000 dilution, Abcam, lot: ab28146), anti-rabbit CYP3A1 (1:1000 dilution, Abcam, lot: ab3572), anti-rabbit BCRP (1:500 dilution, Abcam, lot: ab207732), anti-rabbit MDR1 (1:1000 dilution, Abcam, lot: ab170904), anti-rabbit MRP2 (1:1000 dilution, Sigma-Aldrich, lot: M8316), anti-rabbit OATP2B1 (1:500 dilution, Novus, lot: NBP-1-59811), anti-rabbit OCT1 (1:1000 dilution, Abcam, lot: ab178869), and anti-rabbit PEPT1 (1:500 dilution, Thermo Fisher, lot: PA5-37010) antibodies were used for Western blotting. The total RNA extraction reagent (lot: RC101-01) and the PrimeScript RT reagent Kit (lot: R223-01) were obtained from Vazyme Biotech (Nanjing, China).

### Treatment of animals

This study was approved by the Ethics Committee of Qinghai University (Permit No. 2017-15). All experiments strictly followed the National Institutes of Health Guide for the Care and Use of Laboratory Animals. Male Sprague–Dawley rats (180 ± 20 g) were provided by the Experimental Animal Center of Xi’an Jiaotong University (Xi’an, China) (license No. SCXX [Shaanxi] 2018-001). All rats were maintained in a temperature (23°C ± 2°C)- and humidity (55% ± 5%)-controlled room with a 12-h light/dark cycle. The rats were allowed to acclimatize to the new environment prior to experiments, with access to irradiated standard chow (10 g/100 g) and acidified water (10 ml/100 g) every day.

The rats were randomly divided into plain (Cont-P), high-altitude hypoxic (Cont-H), plain pseudo-germ-free (ABX-P), and hypoxic pseudo-germ-free (ABX-H) groups, with six rats per group. The rats in the Cont-P and ABX-P groups were housed in Xi’an city (altitude: 390 m, PaO_2_: 20 kPa) in the Shaanxi Province of China. The rats in Cont-H and ABX-H groups were transported to Maduo County (altitude: 4300 m, PaO_2_: 12.4 kPa) in the Qinghai Province, China. The rats in ABX-P and ABX-H groups were administered an antibiotic cocktail (vancomycin, 0.5 g/L; neomycin sulfate, 1 g/L; ampicillin, 1 g/L; and metronidazole, 1 g/L) for 7 days prior to experimentation.

Fecal microbiota samples and rat liver tissues were collected from individual rats at 9:00 a.m. on the eighth day and immediately stored at −80°C. All tissues were deposited in liquid nitrogen and then transported to the Research Center for the High Altitude Medicine of Qinghai University. Protein and mRNA expression levels were determined using Western blotting and RT-qPCR, respectively, at Qinghai University.

### Sequencing of the 16S ribosomal RNA gene

Total bacterial DNA of rat fecal samples was extracted using a HiPure Stool DNA Kit according to the manufacturer’s instructions. The 16S rRNA V3–V4 region was amplified by PCR (98°C for 30 s, followed by 30 cycles at 98°C for 10 s, 60°C for 30 s, and 72°C for 30 s, and a final extension at 72°C for 120 s) using the following primers: 341F: 5′-CCTACGGGNGGCWGCAG-3′ and 806R: 5′-GGACTACHVGGGTATCTAAT-3′. The generated amplicons were purified using the AxyPrep DNA Gel Extraction Kit and quantified using the ABI StepOnePlus Real-Time PCR System before sequencing on the Illumina platform. Raw reads of all samples were uploaded to the NCBI Sequence Read Archive database (Accession No. PRJNA835243).

Bioinformatic analysis (OTUs, community composition, indicator species, *α*-diversity, and *β*-diversity) of the raw data was performed using various software tools, including Userach (version 7.0), Krona (version 2.6), Vegan (version 2.5.3), QIIME (version 1.9.1), and Muscle (version 3.8.31).

### Western blotting

The total protein of the liver tissues was extracted, and Western blotting analysis was conducted as described in our previous study (Duan et al., 2021). Antibodies against β-actin, CYP1A2, CYP2B1, CYP2C11, CYP2E1, CYP3A1, BCRP, MDR1, MRP2, OATP2B1, OCT1, and PEPT1 were used. The Amersham imager 600 ECL system (Boston, MA, United States) was used to detect specific protein bands on Western blots.

### RT-qPCR

RNA was isolated from liver tissues, and cDNA synthesis and quantitative RT-qPCR were conducted as described in our previous study (Duan et al., 2021). Primer sequences to amplify β-actin, CYP1A2, CYP2B1, CYP2C11, CYP2E1, CYP3A1, BCRP, MDR1, MRP2, OATP2B1, OCT1, and PEPT1 are shown in Table 1. The 2^−ΔΔCt^ method was used to calculate relative fold changes in the expression.

**TABLE 1:** Primer sequences corresponding to the rat genes examined with a RT-qPCR analysis.

Gene	Oligonucleotide primer sequence (5–3′)	
β-Actin	Forward	TCA​CCA​ACT​GGG​ACG​ATA​TG
	Reverse	GTT​GGC​CTT​AGG​GTT​CAG​AG
CYP1A2	Forward	CCA​CAG​CAC​AAC​GAG​GGA​CAC
	Reverse	GCT​CTG​GGC​GGA​ACA​CAA​AGG
CYP2B1	Forward	TTT​GGT​GGA​GGA​ACT​GCG​GAA​ATC
	Reverse	AGG​AAC​TGG​CGG​TCT​GTG​TAG​TC
CYP2C11	Forward	ACA​ATC​CGC​AGT​CTG​AGT​TTA​CCC
	Reverse	AGC​AGC​AGC​AGG​AGT​CCA​TAC​C
CYP2E1	Forward	CGC​TTC​GGG​CCA​GTG​TTC​AC
	Reverse	GTA​GCA​CCT​CCT​TGA​CAG​CCT​TG
CYP3A1	Forward	CGT​TCA​CCA​GTG​GAA​GAC​TCA​AGG
	Reverse	TTC​TTT​CAC​AGG​GAC​AGG​TTT​GCC
BCRP	Forward	TTA​GGA​CTG​AAG​AGG​ACG​GTG​GAG
	Reverse	TTG​CTA​CAG​ACA​CTA​CGC​TTT​GGC
MDR1	Forward	CTC​GCT​GCT​ATC​ATC​CAC​GGA​AC
	Reverse	CGC​TGA​CGG​TCT​GTG​TAC​TGT​TG
MRP2	Forward	TGG​ATT​CCC​TTG​GGC​TTT​CTT​TGG
	Reverse	AAC​ACG​ACG​AAC​ACC​TGC​TTG​G
OATP2B1	Forward	CTG​TCT​GCC​GCT​ACT​ATG​ACC​ATG
	Reverse	CTC​TGC​TCT​GCT​GCC​TCA​AGA​TG
OCT1	Forward	TGC​CTA​CCT​TCC​TCT​TCC​TGC​TG
	Reverse	GCG​TGG​TTC​TCT​TCT​GGG​ACA​AC
PEPT1	Forward	CTT​CAG​GCA​GGA​TGG​CTT​CTA​ACC
	Reverse	AGC​AAG​GAG​GCG​AAC​AGA​ACA​TAC

### Statistical analysis

Compositional data analysis was performed using R software (version 3.5.1). The *α*-diversity index, indicated species, and functional difference analyses were carried out using the Wilcoxon rank-sum test for comparisons between two groups, and the Kruskal–Wallis H test was used for comparisons among multiple groups. The *β*-diversity analysis was performed using the ANOSIM test. All numerical data are expressed as the mean ± SD. Protein and mRNA expression levels in rats were analyzed using the two-way analysis of variance, and variance non-homogeneity was determined using the Levene’s test. The statistical significance was set at *p* < 0.05.

## Results

### High-altitude hypoxia induces gut microbiota changes

**FIGURE 1:**
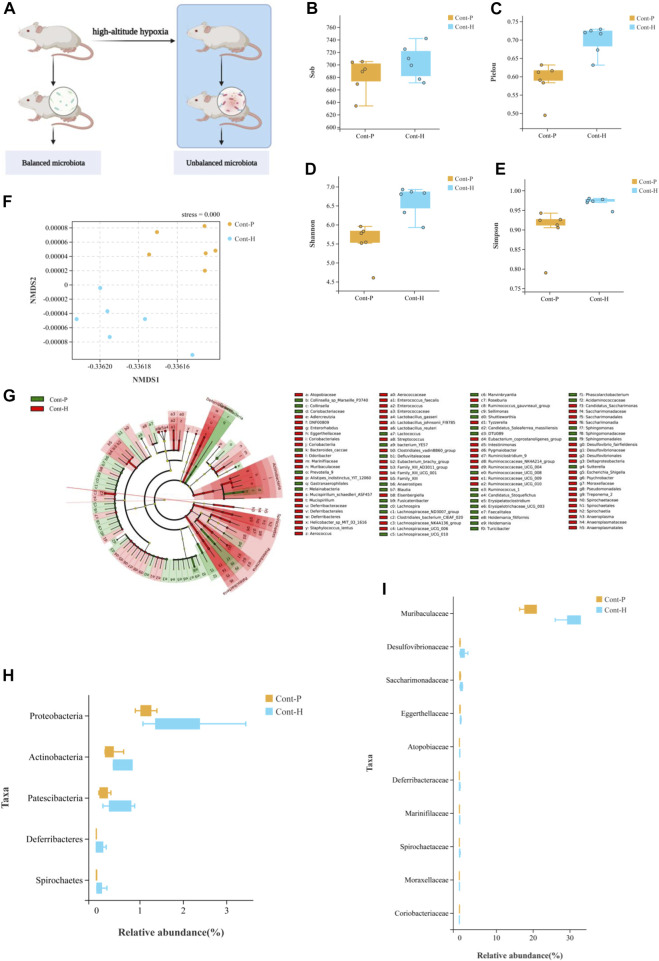
Change in gut microbiota diversity and composition under high-altitude hypoxia. **(A)** Flow chart of experimental animal treatment. **(B–E)** *α*-Diversity analysis of gut microbiota in Cont-P and Cont-H groups. **(F)** *β*-Diversity analysis of gut microbiota in Cont-P and Cont-H groups. **(G–I)** Analysis of differences in the microbiota between Cont-P and Cont-H groups.

To explore the connection between gut microbiota and hypoxia, we established a rat model in a real plateau hypoxic environment and analyzed the fecal gut microbial composition. Hypoxic treatment did not alter the richness of the gut microbiota but did alter its evenness (Figures 1B–E). The *β*-diversity analysis demonstrated that the gut microbiota clustered separately at different altitudes (Figure 1F). Thus, hypoxic treatment induced changes in gut microbial diversity.

The LEfSe algorithm and Mann–Whitney test (Figures 1G–I) analyses were used to characterize changes in the proportions of taxa. Interestingly, hypoxic treatment affected the abundance of taxa. At the phylum level, the Cont-H group had a higher relative abundance of Proteobacteria, Actinobacteria, Patescibacteria, and Spirochetes (*p* < 0.05). At the genus level, the Cont-H group displayed a higher relative abundance of *Lachnospiraceae*_NK4A136_group*, Eubacterium_coprostanoligenes_*group, *Ruminococcaceae_*NK4A214_group, *Candidatus_Saccharimonas*, *Ruminococcaceae_*UCG-009, *Escherichia–Shigella*, *Ruminiclostridium_*9, *Lachnospiraceae_*UCG-006, *Enterorhabdus*, *Pygmaiobacter*, *Ruminococcaceae_*UCG-010, *Tyzzerella*, *Mucispirillum*, *Treponema*_2, *Streptococcus*, and *Psychrobacter* (*p* < 0.05). Thus, hypoxic treatment induced changes in gut microbial composition.

In particular, the proportion of aerobic bacteria was reduced, whereas the proportion of anaerobic bacteria clearly increased in the Cont-H group. Specifically, the compositions of aerobes and anaerobes in the Cont-P and Cont-H groups differed. Based on these results, we conclude that the reduced abundance of aerobic bacteria and the increase in abundance of anaerobic bacteria may be associated with a high-altitude hypoxic environment.

### Reduction in gut microbiota abundance by antibiotics

**FIGURE 2:**
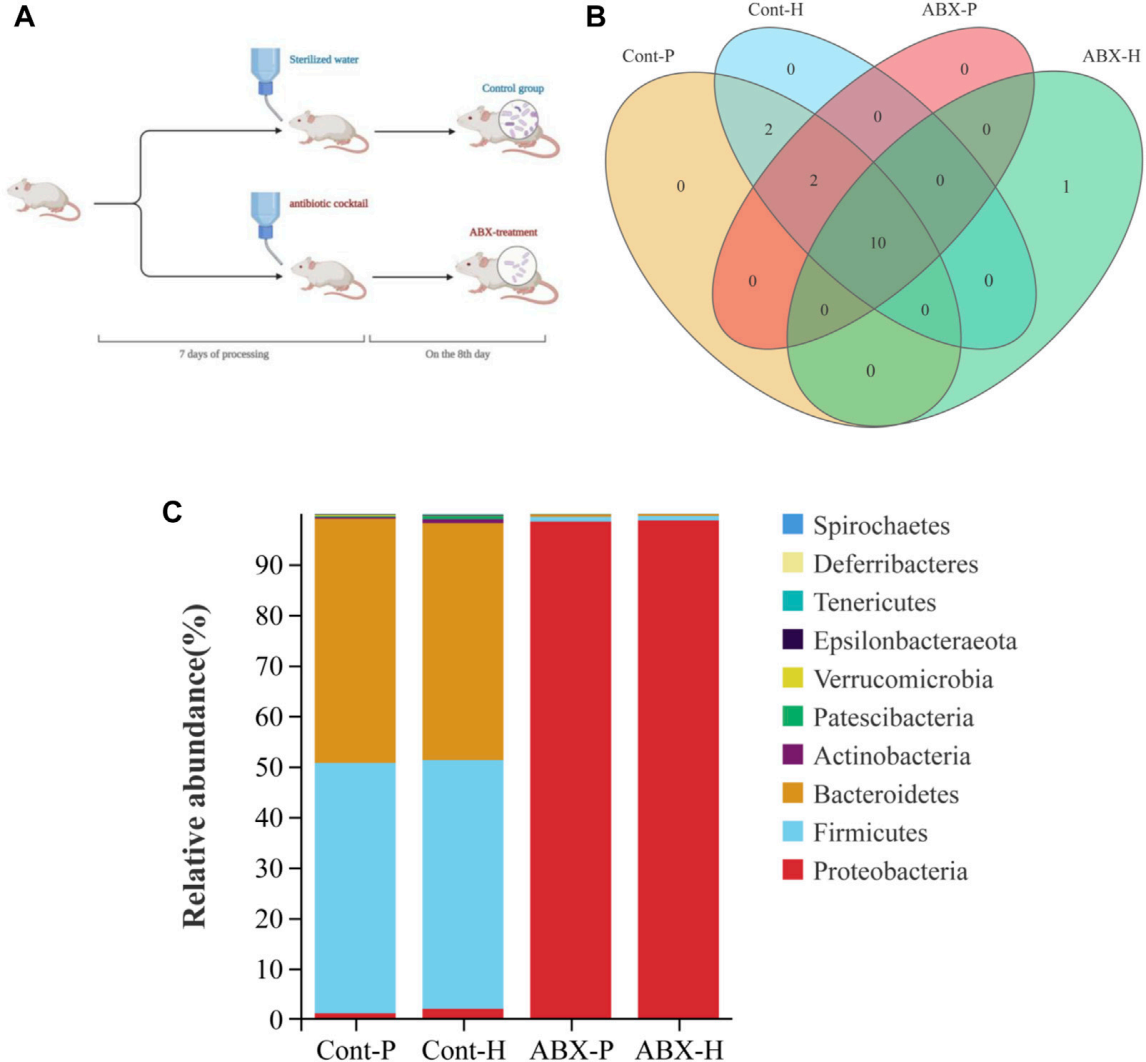
Treatment with the antibiotic cocktail significantly changes in gut microbiota. **(A)** Flow chart of experimental animal treatment. **(B)** Venn diagrams of OTU demonstrating overlap in rats among Cont-P, Cont-H, ABX-P, and ABX-H groups. **(C)** At the level of phylum, the histogram of community structure of gut microbiota in rats among Cont-P, Cont-H, ABX-P, and ABX-H groups.

We investigated the effects of hypoxia on the gut microbiota and the potential factors affecting drug metabolism *in vivo*. First, we attempted to eliminate the gut microbiota of rats through antibiotic treatment. More specifically, treatment with a broad-spectrum antibiotic cocktail for 7 days resulted in a sustained reduction in microbial load (Figure 2B). As expected, 16S rRNA gene sequencing revealed perturbations in the microbiota of rats treated with the antibiotic cocktail for 7 days (Figure 2C). Therefore, the gut microbiota in ABX-treated rats was successfully and homogenously depleted.

### Changes in CYP450 and drug transporter expression mediated by gut microbiota under high-altitude hypoxia

**FIGURE 3:**
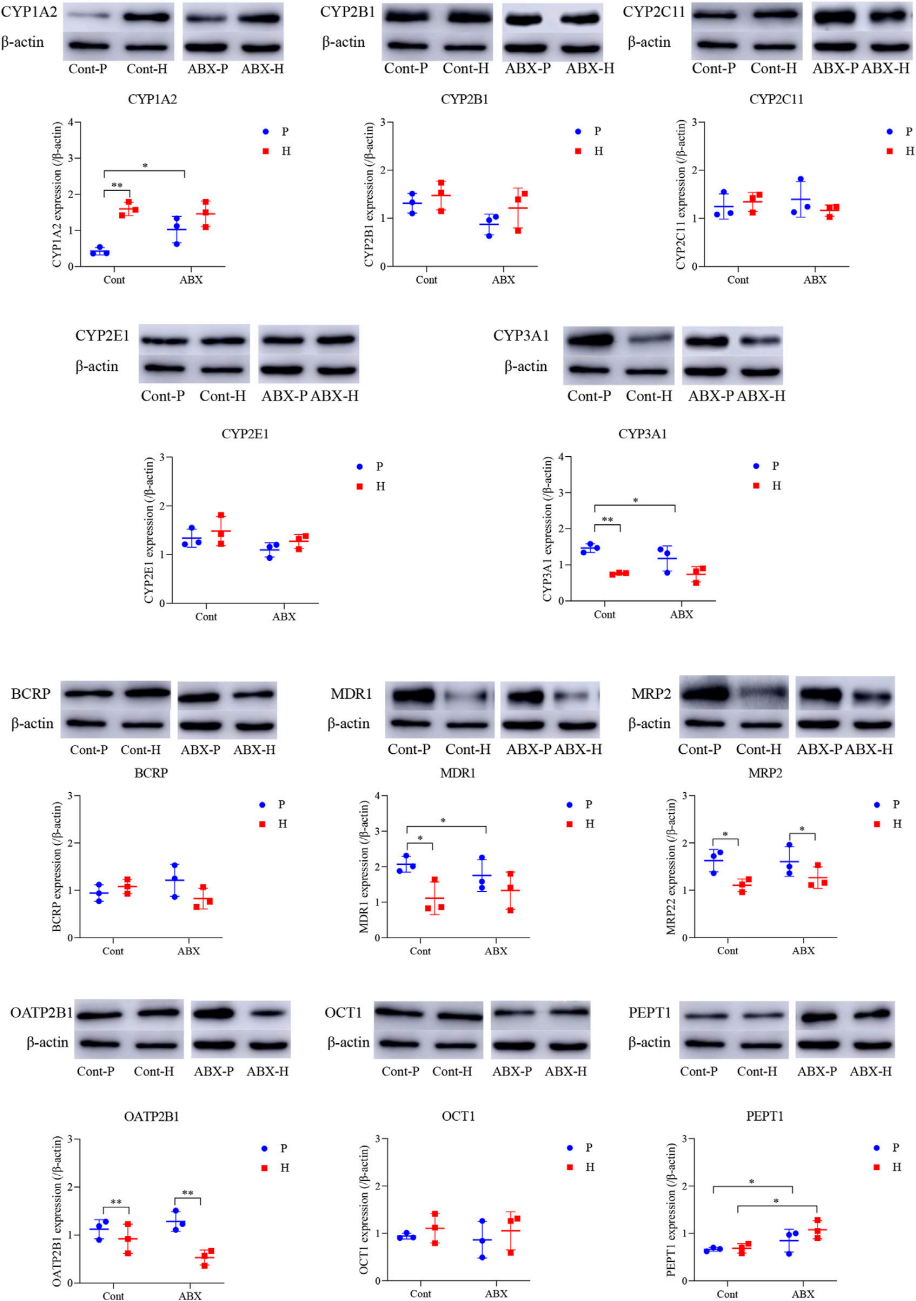
Protein expression of CYP450 and drug transporter in rats among Cont-P, Cont-H, ABX-P, and ABX-H groups. β-Actin expression from the same sample acts as an internal control. Values are expressed as mean ± SD (n = 3), ^*^ *p* < 0.05, ^**^ *p* < 0.01.

Another interesting finding of our study was that CYP450 and drug transporter levels were significantly altered in hypoxia- and ABX-treated rats. MRP2 and OATP2B1 proteins were significantly differentially expressed under high-altitude hypoxia (*p* < 0.05). The protein expression of PEPT1 was significantly increased in the ABX-P and ABX-H groups compared to the Cont-P and Cont-H groups, respectively (*p* < 0.05). The protein levels of CYP3A1 and MDR1 were significantly decreased, whereas the protein level of CYP1A2 expression was significantly increased in the Cont-H group compared to the Cont-P group (*p* < 0.05). Compared to the Cont-P group, the ABX-P group showed a significant decrease in the protein levels of CYP3A1 and MDR1, and a significant increase in CYP1A2 (*p* < 0.05). There was no significant difference in the protein levels of CYP2B1, CYP2C11, CYP2E1, BCRP, or OCT1 in high-altitude hypoxic environments (Figure 3).

**FIGURE 4:**
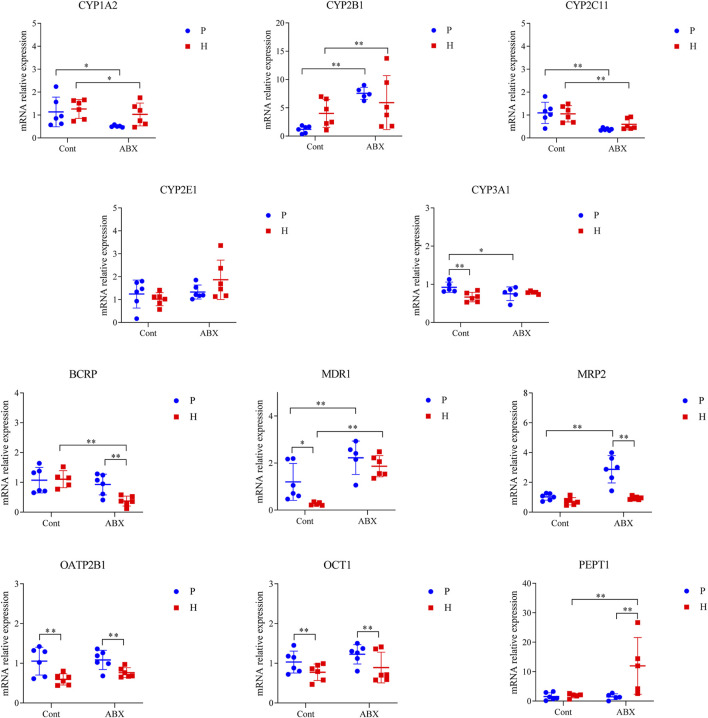
mRNA expression of CYP450 and drug transporter in rats among Cont-P, Cont-H, ABX-P, and ABX-H groups. Values are expressed as mean ± SD (*n* = 6), ^*^ *p* < 0.05, ^**^ *p* < 0.01.

Hypoxia was also associated with reduction in mRNA levels of OATP2B1 and OCT1 (*p* < 0.05). The mRNA expressions of CYP1A2 and CYP2C11 were significantly decreased, whereas the expression of CYP2B1 was increased in ABX-treated groups (*p* < 0.05). Compared to the Cont-P group, the Cont-H group showed a significant decrease in the mRNA levels of CYP3A1 and MDR1 (*p* < 0.05). Compared to the ABX-P group, the ABX-H group showed a significant decrease in the mRNA levels of BCRP and MRP2 expressions, and a significant increase in the mRNA levels of PEPT1 (*p* < 0.05). CYP3A1 mRNA expression was significantly decreased; however, MDR1 and MRP2 mRNA expressions were significantly increased in the ABX-P group compared to the Cont-P group (*p* < 0.05) (Figure 4).

## Discussion

The gut–liver axis plays a pivotal role in various metabolic diseases (Barretto et al., 2021). The liver is an important metabolic and detoxification organ in the body, driving the biotransformation of xenobiotic chemicals and endogenous compounds such as drugs and bile acids, respectively (Desvergne et al., 2006). CYP450 and drug transporter are highly expressed in the liver, are the main factors that regulate drug metabolism, and are mediated by the gut microbiota either directly or indirectly (Willyard, 2018). Therefore, disordered gut microbiota may be involved in drug metabolism by affecting hepatic CYP450 and drug transporter. Our study linked CYP450, drug transporter, gut microbiota, and high-altitude hypoxia, thus highlighting previously ignored aspects of the hypoxia–gut microbiota–CYP450/drug transporter axis, which could have implications on drug metabolism.

In addition to its fundamental function in facilitating the metabolism of endogenous xenobiotics, the gut microbiota helps maintain gut homeostasis (Shreiner et al., 2015). In our study, 16S rRNA gene sequencing analysis revealed the influence of hypoxia on the gut microbiota in terms of the overall bacterial diversity. However, in a study by Zhang et al. (2018), inconsistent results were observed for *α*-diversity. The difference in these results is attributable to the different types of hypoxic treatments and control group settings used. For example, our study focused on a real plateau hypoxic environment rather than simulated hypoxia, and our control group on a low elevation plain, rather than at 2200 m above the sea level. Importantly, a low-pressure oxygen chamber cannot simulate the characteristics of a real hypoxic plateau environment, such as strong radiation, drought, and temperature. A previous study by Ramos-Romero et al. (2020) identified hypoxia-induced changes in the gut microbiota. Furthermore, Li and Zhao (2015) revealed that the gut microbiota differs between Chinese Han people living at low and high altitudes. Notably, our classification analysis of the taxonomic levels of different species also revealed a remarkable difference in gut microbiota composition between animals at the plain and the plateau, thus corroborating the findings of previous studies. Under hypoxic conditions, pathological changes may cause intestinal problems and alter the dynamic balance of the gut microbiota (Xing et al., 2018; Sun et al., 2019; Nijiati et al., 2021). A vast body of literature illustrates that oxygen is associated with gut inflammation and oxidative stress, shaping the balance of the intestinal epithelium between aerobiosis and anaerobiosis (Mu and Zhu, 2019). This fact explains, at least in part, why the gut microbiota is susceptible to hypoxia. Thus, our results quantify the strong influence of geographical background as well as the environment (especially hypoxia) on gut microbial diversity and composition.

CYP450 participates in the metabolism of most drugs, and more important is susceptible to oxygen concentration. In addition, the effect of drug transporters on drug metabolism under high-altitude hypoxia cannot be overlooked. Based on these facts and previous studies, we selected CYP450 (CYP1A2, CYP2B1, CYP2C11, CYP2E1, and CYP3A1) and drug transporters (MDR1, MRP2, BCRP, OATP2B1, OCT1, and PEPT1) as research subjects. Our findings were further confirmed by analysis of the expression of CYP450 and drug transporters under high-altitude hypoxic conditions. Most notably, downregulation of CYP3A1 and MDR1 expressions at both the protein and mRNA levels was observed. Moreover, according to our previous studies, the metabolism of midazolam and rhodamine 123, the corresponding substrates of CYP3A1 and MDR1 respectively, has changed accordingly (Duan et al., 2022). In addition, CYP3A1 and MDR1 participate in the metabolism and transport of over half of currently used pharmacological agents (Takano et al., 2021). As such, the results of this study highlight the potential role of hypoxia on CYP450 and drug transporter, notably CYP3A1 and MDR1, and suggest that the pharmacokinetics of drugs metabolized and transported by these proteins may be affected in hypoxic environments.

In our study, CYP3A1 was one of the most downregulated CYP450 in the gut microbiota under high-altitude hypoxic conditions. We demonstrated that the gut microbiota might be a critical mediator of changes in the CYP3A1 expression. Recently, the gut microbiota has been implicated in drug metabolism, which influences drug efficacy and toxicity (Carmody and Turnbaugh, 2014; Koppel et al., 2017). Our results highlight the potential mechanism underlying CYP450-mediated drug metabolism under hypoxic conditions. Clinically, CYP3A4 metabolizes a large number of drugs, and its activity (induction or inhibition) is regarded as the major factor in drug–drug interactions (Jones et al., 2017; Prueksaritanont et al., 2017). These findings indicate that the gut microbiota, *via* CYP3A1, may partially affect drug metabolism under hypoxic conditions. Whether this finding can guide the rational use of drugs in hypoxic areas remains undetermined.

Under hypoxia, a strong negative correlation was observed between *L. murinus* and the expression level of MDR1. Notably, an increase in MDR1 expression and function induced by *L. murinus* under normal and inflammatory conditions has been reported (Saksena et al., 2010). Interestingly, our results showed that the abundance of *L. murinus* was extremely low under hypoxic conditions. Thus, the decrease in *L. murinus* abundance may contribute to the decreased expression of MDR1 under hypoxia. Furthermore, we identified the gut microbial composition that could induce MDR1 expression under hypoxia, which included the genus *Clostridium*. Altogether, our results show a bidirectional relationship between gut microbiota and MDR1, which mutually affect each other. However, the underlying mechanism was not investigated in this study, and further research is required to address this issue.

Furthermore, the complex mechanism of gut microbiota–mediated CYP450 and drug transporter expression in high-altitude hypoxic environments and the limitations in using a pseudo-sterile rat model constructed with antibiotics require further investigation. Critically, it is necessary to conduct future studies to confirm whether the changes observed in this study regarding the expression changes of CYP450 and drug transporters were a direct result of the gut microbiota, as opposed to potential hypoxia-induced physiological changes leading to their altered expression.

**FIGURE 5:**
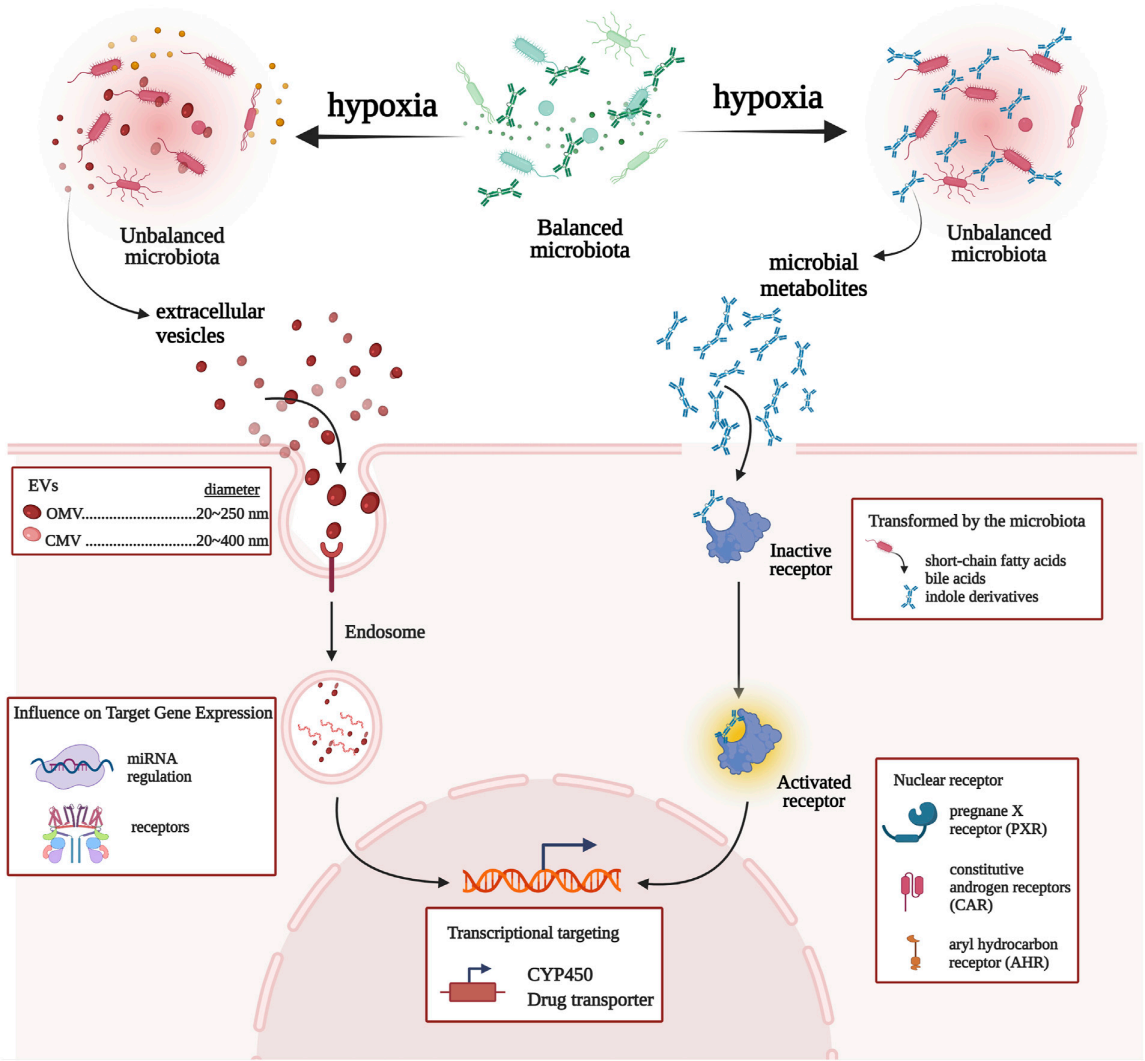
Network view of the interactions between gut microbiota and CYP450/drug transporter under high-altitude hypoxia.

Recently, the mechanisms linking gut microbial communities with drug metabolism have provided prominence to microbiota and CYP450/drug transporter as pivotal participants. In this study, we propose a microbiota–CYP450/transporter axis functioning in high-altitude hypoxic environments (Figure 5). ① Microbiota-derived metabolites activate receptors and signaling pathways, which induce CYP450/drug transporter expression. ② Gut microbiota is able to communicate with the host through the secretion of extracellular vesicles, which penetrate host intestinal barriers, enter the systemic circulation, and then regulate CYP450/drug transporter expression, thereby regulating drug metabolism. Therefore, the gut microbiota may be a key regulator of drug metabolism and should be a topic of research on drug metabolism in high-altitude hypoxic environments.

## Conclusion

We have identified changes in gut microbial diversity and composition in high-altitude hypoxic environments. Moreover, we confirmed that hypoxia changes the expression of CYP450/drug transporter and illustrated that the gut microbiota–CYP3A1/MDR1 interaction affects drug metabolism. We proposed a new perspective for drug metabolism and gut microbiota and a potential new mechanism for gut microbiota–targeted drug interactions in high-altitude hypoxic environments.

## Data Availability

The datasets presented in this study can be found in online repositories. The names of the repository/repositories and accession number(s) can be found at: NCBI BioProject, PRJNA835243.
